# An Introduction to the China Rice Functional Genomics Program

**DOI:** 10.1002/cfg.147

**Published:** 2002-04

**Authors:** Yongbiao Xue, Zhihong Xu

**Affiliations:** 1 Institute of Genetics and Developmental Biology The Chinese Academy of Sciences Beijing 100080 China; 2 The Chinese Academy of Sciences Beijing 100864 and Beijing University Beijing 100871 China

## Abstract

The China Rice Functional Genomics Program (CRFGP) was initiated in 1999 by the Ministry of Science and Technology of China under the National Basic Sciences Initiative and was expected to last for an initial period of five years. The CRFGP involves 20 research groups from the Chinese Academy of Sciences and some major universities and focuses on the identification of genes controlling flowering, plant architecture, fertility, reproduction, metabolic controls and stress responses in rice through a combinatorial approach based on genetics, molecular biology and functional genomics as well as the generation of intellectual properties related to crop breeding and improvements. We will briefly describe the mission of the CRFGP as well as its recent progress.


**Comparative and Functional Genomics**
*Comp Funct Genom* 2002; **3**: 161–163.
Published online 12 March 2002 in Wiley InterScience (www.interscience.wiley.com). **DOI:** 10.1002/cfg.147

**Conference Review**

### An introduction to the China Rice Functional Genomics Program

Yongbiao Xue[1]* and Zhihong Xu[2]

[1] *Institute of Genetics and Developmental Biology, The Chinese Academy of Sciences, Beijing 100080, China*
[2] *The Chinese Academy of Sciences, Beijing 100864 and Beijing University, Beijing 100871, China*

**Correspondence to:*
*Institute of Genetics and Developmental Biology, The Chinese Academy of Sciences, Beijing 100080, China.*
*E-mail: ybxue@genetics.ac.cn*

Received: 28 January 2002
Accepted: 30 January 2002

### Abstract

**The China Rice Functional Genomics Program (CRFGP) was initiated in 1999 by the Ministry of Science and Technology of China under the National Basic Sciences Initiative and was expected to last for an initial period of five years. The CRFGP involves 20 research groups from the Chinese Academy of Sciences and some major universities and focuses on the identification of genes controlling flowering, plant architecture, fertility, reproduction, metabolic controls and stress responses in rice through a combinatorial approach based on genetics, molecular biology and functional genomics as well as the generation of intellectual properties related to crop breeding and improvements. We will briefly describe the mission of the CRFGP as well as its recent progress.** **Copyright © 2002 John Wiley & Sons, Ltd.**

**Keywords:** **Rice; agronomic traits; functional genomics; China**

Apart being one of the most important food crops for over half the world's population, rice (*Oryza sativa* L.) serves as a major model system in cereal genome research due to its relatively small genome size; estimated at about 430 mega bases (Mb) [1], its relative ease of transformation compared to the other major cereals, and its genetic synteny with other cereal genomes. In addition, an international rice genome sequencing project (IRGSP) is expected to sequence the entire rice genome before the end of 2002 [10]. Two private companies, Monsanto and Syngenta, have announced that they have a working draft, and a virtually completed map, of the rice *O. sativa* spp. *japonica* rice var. Nipponbare genome, respectively [9,3]. Recently, a working draft from another rice subspecies (*O. sativa* spp. *indica* rice var. 9311) has been produced by a group of Chinese scientists [11].

In addition to supporting a rice genome sequencing program started in 1998, the Ministry of Science and Technology of China began the China Rice Functional Genomics Program (CRFGP) in 1999 under the National Basic Sciences Initiative in order to discover genes essential for superior agronomic performance of crops. The CRFGP was expected to last for a first period of five years. A total of 20 research groups from the Chinese Academy of Sciences and some major universities in China are participating in the program. The program focuses on the identification of genes related to flowering, plant architecture, fertility, reproduction, metabolic control and stress responses in rice through a combinatorial approach based on genetics, molecular biology and functional genomics. The program also aims to generate intellectual property relating to genetic resources for rice functional genomics and crop improvements.

The program has been organized into a total of six projects. The respective research targets for the initial five years, and the progress made so far by each are briefly described below.

### Creation of a mutant library

The exploitation of genetic variations is crucial for understanding the function of genome sequences. Two widely used strategies to generate genetic variations in plants are chemical mutagenesis and inactivation of gene function by insertion of T-DNA from *Agrobacterium* or transposable elements, for example, Ac/Ds from maize. Several T-DNA and transposon-tagging populations of rice have been generated [2,6,5,7]. The project aim was

Copyright © 2002 John Wiley & Sons, Ltd.

to generate a population of 3000 molecularly characterized *Ds* transposant lines and 500 EMS- or radiation-induced mutant lines. To create a mutant population, a construct containing Ac/Ds in conjunction with an enhancer trapping strategy is being used to transform rice (*O. sativa* ssp. *japonica* var. Zhonghua 11). So far, over 3000 Ac/Ds lines have been obtained and a few hundred insertion sites have been mapped on the rice chromosomes. Mutants affecting several aspects of rice growth and development related to important agronomic traits, and the genes affected, are being characterized and cloned.

### Expression profiling and discovery of genes related to key agronomic traits

The project planned to obtain >20 000 Uni-ESTs (unique-expressed sequence tags) of rice for cDNA microarray-based expression profiling of genes related to biological processes of interest. To prepare a cDNA-microarray, about 110 000 ESTs from several cDNA libraries constructed from *indica* rice tissues under normal or stressed conditions have been sequenced and over 10 000 unique cDNAs identified. The unique cDNAs have been microarrayed for gene profiling analysis and clusters of genes responsive for drought stresses, and genes specific for developmental stages are being characterized. In addition, a map-based gene cloning strategy has been used to isolate several key genes affecting plant growth and development, such as genes with roles in tillering and plant architecture.

### Development of a TAC-based gene cloning system

Although map-based cloning is feasible, and several important rice genes have been isolated accordingly, it is still a time-consuming task to perform. To speed up cloning of mutated genes and make better use of available genome sequence, the project aimed to establish a platform dealing with large DNA fragment cloning and transfer, based on TAC (transformation-competent artificial chromosome) vectors [8]. TAC vectors for rice transformation, and several TAC libraries containing rice genomic DNA, have been constructed and are being used to build up physical maps for several genes controlling wide-compatibility, temperature-sensitive male sterility and fertility restorations used for hybrid seed production. A few candidate genes have been identified.

We are also generating a TAC-based rice genome physical map by sequencing TAC clone ends and aligning them to known rice genome sequence. Once this is done, TAC clones covering the genetic map position of a mutated gene could be used for complementation experiments to quickly pin down the target gene-containing TAC clone, providing an access to the target gene. This could bypass the use of large segregating populations and fine-mapping of target genes using the conventional map-based gene cloning procedure and therefore saves considerable time and resources.

### Functional genomics of rice transcription factors

Transcription factors are key regulators for growth and development as well as physiological and biochemical processes. The project plan was to complete a genome-wide identification and functional analysis of 1–2 classes of transcription factors, especially those involved in controlling grain quality and yield in rice. Hundreds of transcription factors, including MADS, MYB and HB, have been isolated and are being used to transform rice plants. A wide spectrum of phenotypes is expected with possible applications in rice breeding programs.

### Production of RicegeneDB

The project aim was to develop an integrated database for rice gene information from CRFGP and other related projects worldwide, and bioinformatic tools for data analysis and management. Currently, the database contains rice mutant descriptions and EST data. We are also planning to integrate results from our microarray analyses and TAC end sequencing into the database. The results from CRFGP are updated from time to time and can be accessed at the web site (**http://www.rifgp.ac.cn**).

### Development of transgenic rice lines for better agronomic performance

To build up a pipeline from genes to traits, the project is developing a platform for genetic improvements of key agronomic traits including a

Copyright © 2002 John Wiley & Sons, Ltd. 

robust transformation technology and desired expression systems. Using an antisense *Waxy* gene in conjunction with its own promoter, we have developed transformation procedures for most cultivated rice lines in China using *Agrobacterium*, and the obtained transgenic lines are stable over several generations in field tests. Several of the genes discovered through this program are currently being tested to improve rice yield and grain quality.

### Conclusion

In conclusion, making sense of rice genes as well as its genome sequence clearly requires a well-coordinated effort of the international community [4] as other functional genomics programs. Therefore, the CRFGP is open to international collaborations and willing to share our data with the research community to realize the benefits of rice functional genomics for improved rice and other crops for world food security.

### Acknowledgement

The CRFGP is funded by the Ministry of Science and Technology of China (G1999011600). The initial preparation for the program was supported by the Chinese Academy of Sciences. The efforts by all the scientists involved in the CRFGP, in particular, Jiayang Li, Yaoguang Liu, Jingliu Zhang, Da Luo, Hongwei Xue, Kang Chong, Hai Huang and Guohua Liang, are greatly appreciated. We also thank Bin Han for sequencing and Qifa Zhang for sharing rice ESTs.

### References

1. Arumuganathan K, Earle ED. 1991. Nuclear DNA content of some important plant species. *Plant Mol Biol Rep* **9**: 208–218.
2. Chin HG, Choe MS, Lee SH, *et al*. 1999. Molecular analysis of rice plants harboring an Ac/Ds transposable element-mediated gene trapping system. *Plant J* **19**: 615–623.
3. Davenport RJ. 2001. Syngenta finishes, consortium goes on. *Science* **291**: 807.
4. Fischer KS, Barton J, Kush GS, Leung H, Cantrell R. 2000. Collaborations in rice. *Science* **290**: 279–280.
5. Greco R, Ouwerkerk PB, Taal AJ, *et al*. 2001. Early and multiple Ac transposition in rice suitable for efficient insertional mutagenesis. *Plant Mol Biol* **46**: 763–764. Erratum **46**: 215–227.
6. Jeon JS, Lee S, Jung KH, *et al*. 2000. T-DNA insertional mutagenesis for functional genomics in rice. *Plant J* **22**: 561–570.
7. Kohli A, Xiong J, Greco R, Christou P, Pereira A. 2001. Tagged Transcriptome Display (TTD) in indica rice using Ac transposition. *Mol Genet Genomics* **266**: 1–11.
8. Liu YG, Shirano Y, Fukaki H, *et al*. 1999. Complementation of plant mutants with large genomic DNA fragments by a transformation-competent artificial chromosome vector accelerates positional cloning. *Proc Natl Acad Sci U S A* **96**: 6535–6540.
9. Pennisi E. 2000. Stealth genome rocks rice researchers. *Science* **288**: 239–241.
10. Sasaki T, Burr B. 2000. International Rice Genome Sequencing Project: The effort to completely sequence the rice genome. *Curr Opin Plant Biol* **3**: 138–141.
11. Yu J, Hu SY, Wang J, *et al*. 2001. A working draft and initial analysis of the rice genome (*Oryza sativa* ssp. *indica*). *Chinese Sci Bull* **46**:1397–1341.

Copyright © 2002 John Wiley & Sons, Ltd. 
